# Stimuli Characteristics and Psychophysical Requirements for Visual Training in Amblyopia: A Narrative Review

**DOI:** 10.3390/jcm9123985

**Published:** 2020-12-09

**Authors:** Carlos J. Hernández-Rodríguez, David P. Piñero, Ainhoa Molina-Martín, León Morales-Quezada, Dolores de Fez, Luis Leal-Vega, Juan F. Arenillas, María Begoña Coco-Martín

**Affiliations:** 1 Group of Optics and Visual Perception, Department of Optics, Pharmacology and Anatomy, University of Alicante, San Vicente del Raspeig, 03016 Alicante, Spain; carlosj.hernandez92@gmail.com (C.J.H.-R.); ainhoa.molina@ua.es (A.M.-M.); dolores.fez@ua.es (D.d.F.); 2 Clinical Optometry Unit, Department of Ophthalmology, Vithas Medimar International Hospital, 03016 Alicante, Spain; 3 Department of Physical Medicine and Rehabilitation, Spaulding Rehabilitation Hospital, Harvard Medical School, Boston, MA 02215, USA; jmorales@neuromodulationlab.org; 4 Group of Applied Clinical Neurosciences and Advanced Data Analysis, Neurology Department, Faculty of Medicine, University of Valladolid, 47005 Valladolid, Spain; luis.leal.vega.1213@gmail.com (L.L.-V.); juanfarenillas@gmail.com (J.F.A.); mbegococom@gmail.com (M.B.C.-M.); 5 Stroke Unit and Stroke Program, Department of Neurology, Universitary Hospital, University of Valladolid, 47003 Valladolid, Spain

**Keywords:** amblyopia, vision therapy, visual acuity, contrast sensitivity, perceptual learning, dichoptic training

## Abstract

Active vision therapy using perceptual learning and/or dichoptic or binocular environments has shown its potential effectiveness in amblyopia, but some doubts remain about the type of stimuli and the mode and sequence of presentation that should be used. A search was performed in PubMed, obtaining 143 articles with information related to the stimuli used in amblyopia rehabilitation, as well as to the neural mechanisms implied in such therapeutic process. Visual deficits in amblyopia and their neural mechanisms associated are revised, including visual acuity loss, contrast sensitivity reduction and stereopsis impairment. Likewise, the most appropriate stimuli according to the literature that should be used for an efficient rehabilitation of the amblyopic eye are described in detail, including optotypes, Gabor’s patches, random-dot stimuli and Vernier’s stimuli. Finally, the properties of these stimuli that can be modified during the visual training are discussed, as well as the psychophysical method of their presentation and the type of environment used (perceptual learning, dichoptic stimulation or virtual reality). Vision therapy using all these revised concepts can be an effective option for treating amblyopia or accelerating the treatment period when combining with patching. It is essential to adapt the stimuli to the patient’s individual features in both monocular and binocular training.

## 1. Introduction

Amblyopia is a visual developmental disorder consisting of a reduced best-corrected visual acuity in one or, rarely, both eyes without the presence of any ocular pathology. Etiology is often a high refractive error, anisometropia, strabismus, or a combination of these factors. Less common is deprivation amblyopia, which is caused by a pathology that avoids the eye stimulation during childhood and causes a severe visual deficit [1,2]. The prevalence of amblyopia in childhood is approximately between 1 and 3%, although these values differ among authors [3,4]. In amblyopia, the loss of visual acuity and the presence of many other monocular and binocular visual deficits are the consequence of the anomalies in the visual pathway of the amblyopes, mainly in the striate and extra-striate cortex [5]. Amblyopic eyes showed worse neural adaptation in V1, V2, V3, V3a, Vp and V4, that is, a reduced cortical automation after a repeated visual task compared with fellow eyes, and a decreased neural response assessed by functional magnetic resonance imaging (fMRI) [6] as well as a worse effective connectivity between the implied brain regions, which is correlated with the reduced visual acuity [7].

Over the last few years, vision therapy has been suggested to be an effective option to promote visual rehabilitation and even to accelerate recovery when combined with patching [8]. New trends in computer-based active vision therapy have been developed for amblyopia treatment, such as the use of perceptual learning environments [9,10,11], dichoptic stimulation [12,13,14] and binocular training [15,16]. These video games use visual and perceptual tasks, such as orientation discrimination or letter recognition, among others, to cause a response in neuro-modulatory pathways and the enhancement of attentional skills, according to neurophysiological studies [17,18]. These options may support and optimize treatments with spectacles, patching and penalization [19,20].

One of the critical issues when active vision therapy is programmed using perceptual learning and/or a dichoptic or binocular environment is the type of stimuli and the mode and sequence of presentation that should be used. Additionally, depending on the treatment phase, stimuli and environments are specifically selected for promoting visual deficit recovery in a monocular phase, or improving interocular fusion and stereopsis in the binocular phase. Different approaches are commercially available in the form of adaptative software to perform the training at home with online control of the compliance by the practitioner. However, more research is needed on the adequacy of the visual stimuli used for visual training and which psychophysical method would be the most appropriate for the presentation of such stimuli. The aim of the current review article is to gather worthy information on all the main characteristics of the stimulus and the psychophysical method that should be used in amblyopia, depending on the baseline characteristics of the patient, to provide researchers and clinicians a useful guideline for optimizing active vision therapy programs in amblyopia.

## 2. Experimental Section

A search was carried out in September 2019 using a free language strategy in PubMed. The keywords used were related to neural mechanism and stimulus in amblyopia, such as “crowding”, “masking”, “interocular suppression” and “stimulus”, among others. The inclusion criteria were articles which analyze visual deficits, stimuli and psychophysics methods in amblyopia. Neither temporal nor type-of-articles restrictions were applied, including clinical trials, case series, non-controlled comparative studies, and case reports. The exclusion criteria were studies with animals, with no neural perspective or focused on patching treatment.

The search strategy used was the following:

#1. Neural mechanism *
#2. Crowding
#3. Masking
#4. Stereopsis
#5. Visual acuity
#6. VA
#7. Contrast senstiviy
#8. Interocular suppression
#9. Visual deficit *
#10. #1 OR #2 OR #3 OR #4 OR #5 OR #6 OR #7 OR #8 OR #9
#11. amblyopia
#12. anisometropic amblyopia
#13. strabismic amblyopia
#14. lazy eye
#15. #11 OR #12 OR #13 OR #14

## 3. Results

One hundred and forty-three articles were obtained in the first search for documentary support, but only those which met the inclusion criteria were selected. Then, a manual search was performed based on the bibliography of the articles selected. The results were organized in structured sections for a better understanding of the concepts described and an appropriate follow-up of the reader.

### 3.1. Visual Deficits in Amblyopia

For an appropriate treatment, the stimuli used for visual training should be scientific-based and adapted to the specific characteristics of the amblyopic visual system. Thus, a good comprehension of the neural mechanisms and visual deficits of amblyopia is needed.

#### 3.1.1. Crowding, Flankers and Masking

Crowding is one of the most important visual deficits in amblyopia and is described by Levi as a “deleterious influence of nearby contours of visual discrimination, is a form of inhibitory interaction which is ubiquitous in spatial vision” [21]. Crowding is also present in the normal population when the target is surrounded by other stimuli called flankers, with the consequent impairment of the recognition of an object in a clutter [22]. In the peripheral vision of non-amblyope subjects, a letter becomes unrecognizable when there are other stimuli around it, despite both target and flankers being clearly separated in the retinal point spread function (PSF), this being dependent on target eccentricity [23,24]. In addition, in the foveal vision of the non-amblyope subject, crowding can occur when the target–flankers distance is less than 4–6 min of arc [22,25,26]. In amblyopes, crowding occurs with larger distances between targets and flankers, and is related to spatial frequency and target size [27].

Crowding affects many visual tasks such as letter recognition [22,23,24], orientation discrimination [28,29], vernier visual acuity [30] and stereoacuity tasks [31]. Furthermore, crowding seems to be the result of a global visual deficit in amblyopia [21,32,33], although the values of visual acuity (VA), contrast sensitivity and stereoacuity may not have a large impact on it [27].

Flankers are the objects located around the target that impair the target perception due to crowding. In strabismic and mixed amblyopia, which present both anisometropia and strabismus, flankers reduce VA [34]. According to previous authors, when the flankers are complex stimuli, such as letters, the correct term is crowding, but when flankers are sidebars, the term contour interaction should be used [22].

Finally, masking is the impairment of target perception due to an overlapped element called a mask. For people with amblyopia, obtaining the information of the target from an array of stimuli is harder than it is for normal subjects. Repeated practice of a masking task leads to improvements in target detection time in non-amblyopic subjects [35], as well as in crowded and uncrowded visual acuity in amblyopes [26]. Therefore, adding masking tasks to vision therapy might be interesting for future research.

From a neuronal perspective, cortical neuron insufficiency, elevated cortical noise, and abnormal lateral interactions in V1 seem to be related with spatial processing deficits in amblyopic eyes [33].

#### 3.1.2. Contrast Sensitivity Reduction

Contrast sensitivity is the ability to detect differences in contrast luminance at different spatial frequencies of a grating. Amblyopic eyes can present decreased contrast sensitivity at high spatial frequencies or even at all the spatial frequencies, which is the consequence of alterations in the lateral geniculate nucleus and visual striate cortex [36]. Specifically, there are some differences according to its etiology. In anisometropic amblyopia, a significant decrease in global contrast sensitivity compared with normal subjects is commonly reported, while strabismic amblyopes can show normal or increased low frequency contrast sensitivity [37]. It is interesting that a loss in contrast sensitivity could persist despite the recovery of the visual acuity after amblyopia treatment. However, several authors reported improvements in contrast sensitivity after vision training with perceptual learning in amblyopic subjects [11,38,39]. Thus, amblyopia management should involve contrast sensitivity tasks for a global approach [38], with the aim of obtaining similar values in both eyes and facilitating binocular vision.

#### 3.1.3. Stereopsis Impairment

Stereopsis or stereoacuity is the perception of three-dimensionality because of the cortical combination between the images from each eye. For obtaining correct stereoscopic perception, both eyes should have an adequate and similar visual acuity and contrast sensitivity in the presence of ocular alignment [40]. Therefore, stereopsis is one of the main features that should be assessed in the clinical management of amblyopia, since it usually is decreased or absent due to the differences in the perceived images between the amblyopic and the fellow eye. In anisometropic amblyopia, stereopsis is reduced, although it depends on the degree of anisometropia and the loss of visual acuity in the amblyopic eye. In strabismic or mixed amblyopia with constant deviation greater than 12 prism diopters, subjects are stereoblind due to the lack of bifoveal fixation [41]. Stereopsis is important for appropriate performance in activities such as driving, sports or hand-to-eye coordination tasks, although further research is needed to understand more about how different values of stereopsis interfere with daily activities [32]. In accordance with the aim of this review, it is relevant to note that during the treatment of amblyopia, stereopsis should be assessed and trained, since some authors suggested that vision therapy can also improve stereoacuity [16,42,43,44]. Thus, designing exercises with specific stimuli for stereopsis training assumes great significance as part of the treatment in amblyopic subjects.

Recent evidence from functional magnetic resonance imaging (fMRI) studies on the cortical processing of vision in amblyopia shows that, during amblyopic eye stimulation, not only do primary and secondary visual areas present reduced activation when compared to fellow-fixing eye stimulation, but so too do higher-level visual areas, such as the parieto-occipital and temporal cortex. These findings could explain binocular vision deficits in amblyopic patients [45].

### 3.2. Visual Stimuli Used for Vision Training in Amblyopia

There are many types of stimuli which are used in vision training for amblyopia. For example, video games or films are usually used in published studies, commonly in dichoptic training. Despite the fact that they seem to be effective, they also have some features which could be a limitation. Some of these treatments are based on dichoptic training or monocular stimulation, and use eye–hand coordination games [12,14,46] and popular films [47] which are not specifically designed for treating amblyopia, or do not adjust the difficulty of the training to the patient’s progress. However, there are four types of stimuli which have shown to be effective in clinical research that can be easily added to the amblyopia training software, with the possibility of modification according to the patient’s evolution, and these are letter optotypes [48,49], Gabor’s patches [9,48,49,50,51,52], Vernier’s stimuli [53] and random-dot stereograms [16,54] (Table 1).

#### 3.2.1. Letter Optotypes

**Figure 1:**
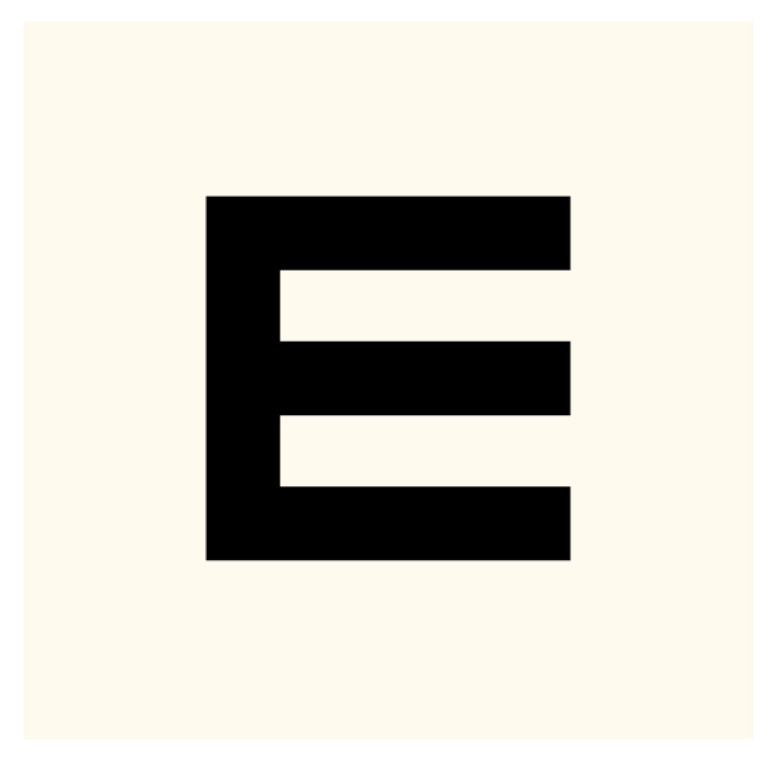
Letter optotype: Snellen E.

As aforementioned, letter recognition is affected by crowding and interaction contours in amblyopia [55], but its use in visual training using perceptual learning approaches can be useful, since many of the parameters of these optotypes can be modified, such as size, contrast sensitivity, presence or absence of flankers, orientation, or motion (Figure 1). For this reason, visual training based on letter optotypes has been used in several scientific articles [56,57].

#### 3.2.2. Gabor’s Patches

**Figure 2:**
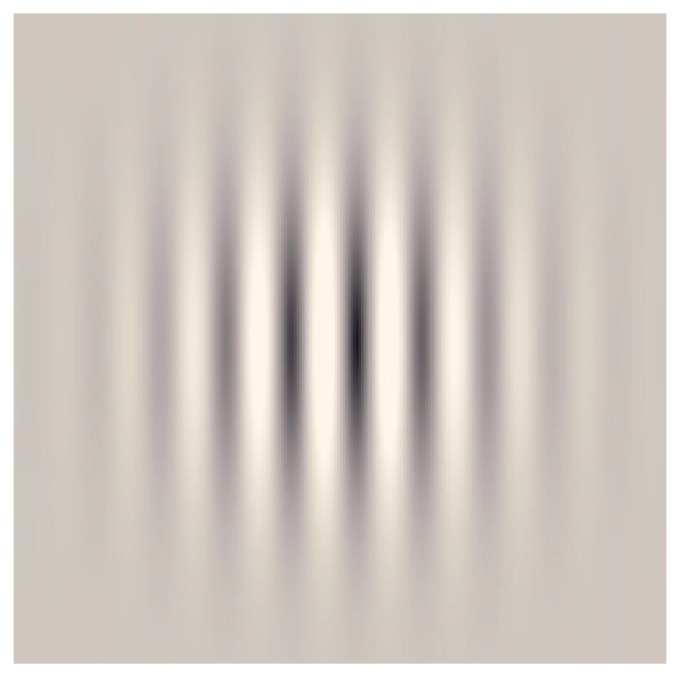
Gabor’s patch.

These are sinusoidal gratings with a Gaussian envelope, which also are commonly used in amblyopia studies, since experimental research demonstrated that gratings with a neutral background can cause selective cortical responses for orientation and contrast, which additionally correlates with fMRI findings (Figure 2) [58,59]. Amblyopia affects high spatial frequency, contrast sensitivity and orientation discrimination, among others visual deficits. Therefore, Gabor´s patches also can be used in perceptual learning, since they allow clinicians to apply many visual tasks, adapting contrast, spatial frequency, size, and other parameters according to each individual case [9,48,49,50,51,52,60].

#### 3.2.3. Random-Dot Stereograms

**Figure 3:**
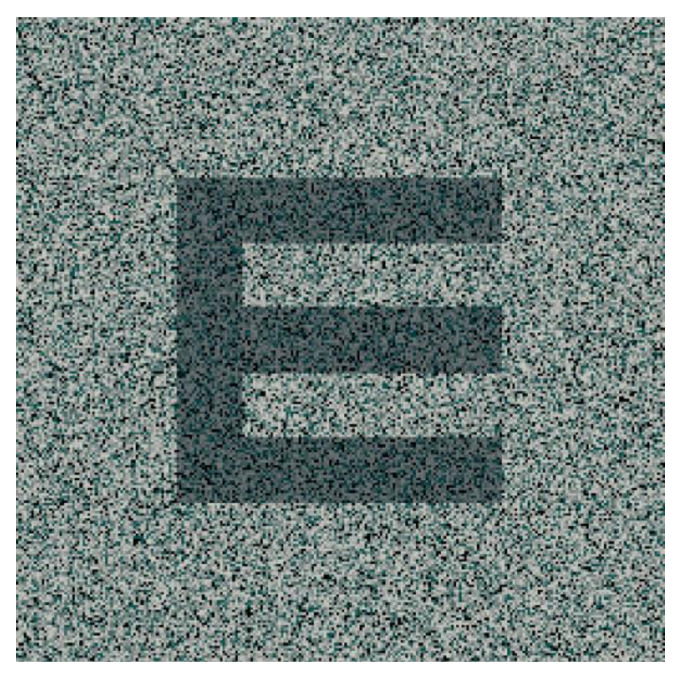
Example of a random-dot stimulus. The dark “E” represents the section of the image that is perceived in depth when seen by subjects with an appropriate binocular vision.

This stimulus consists of an array of dots randomly presented to the subjects for specific stereoscopic perception stimulation (Figure 3). Usually, random-dot stimuli are used for stereoacuity assessment in clinical practice, but recent published works showed their application also in improving stereopsis values in amblyopia [16,54]. The use of a random-dot stereogram entails a cortical activation in the early visual cortex, particularly with mixed-polarity stimulus [61], and a small but significant activation of the pupil and accommodation responses [62]. Additionally, the optimal size of the dots should be considered for well-designed random-dot stimuli, since the literature suggests that larger dots implicate better stereo-perception [63].

#### 3.2.4. Vernier’s Stimulus

**Figure 4:**
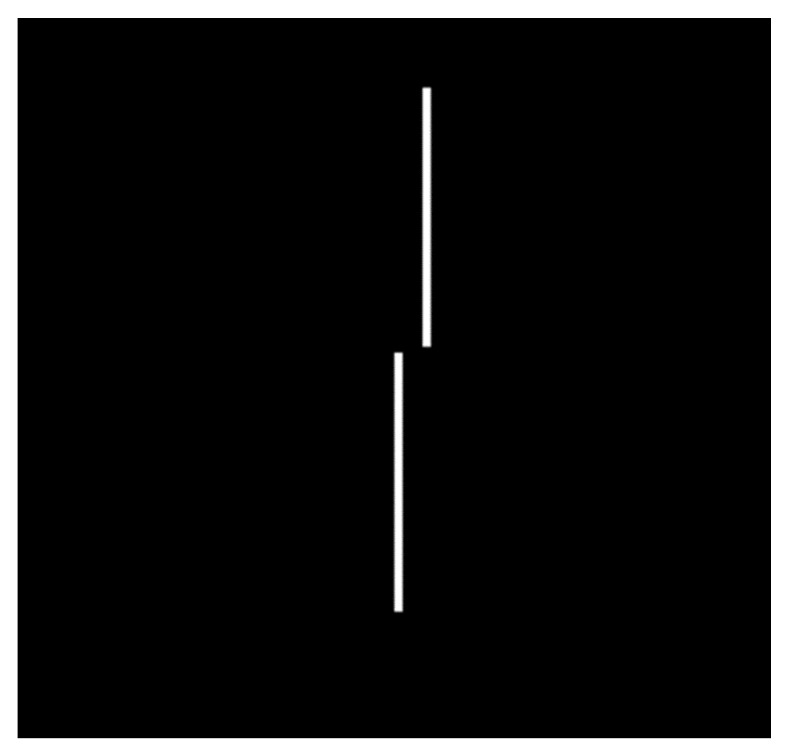
Vernier’s stimulus.

Vernier’s stimuli are based on the perception of the continuity of a lineal stimulus, and are related with the concept of visual hyperacuity, which also is decreased in amblyopia [64]. The less separate the lineal stimuli are, the more difficult it is to perceive discontinuity for amblyopic subjects (Figure 4).

### 3.3. Parameters That Can Be Modified in the Visual Stimuli

There are many parameters that should be considered when performing visual training to treat amblyopia. For such purpose, the characteristics of stimuli that are described below should be considered due to their relationship with the neural mechanism of amblyopic subjects.

#### 3.3.1. Spatial–Temporal Frequency

All types of amblyopes show worse contrast sensitivity for high spatial frequencies [1], which can be related with a worse response to orientation tasks with the increase in spatial frequency. In 1977, Levi and Harwerth [65] reported that the reduction in contrast sensitivity in anisometropic and strabismic amblyopes was due to neural mechanisms, since optical factors and eccentric fixation were dismissed. This deficit was significant for both long and short stimulus durations. In anisometropic amblyopia, a slight decrease could also be observed in the low and middle frequencies, although this can be partially attributed to optical factors according to some authors [66]. These findings are also reported in myopic anisometropic amblyopia [67]. Furthermore, in the case of meridional amblyopia, the visual deficit is dependent on the cylinder axis [68,69]. In strabismic amblyopia, the loss in contrast sensitivity is more significant and can affect mainly high frequencies or even all spatial frequencies [70].

Amblyopes also show temporal frequency deficits. Yang et al. [71] reported a larger temporal discrimination threshold in amblyopic eyes in 5 and 10 Hz stimuli compared with the fellow eyes of the same subject in anisometropic and strabismic amblyopia, and between fellow eyes and normal eyes only in strabismic amblyopes. This temporal frequency deficit did not show a correlation with logarithmic minimum angle of resolution (logMAR) visual acuity, although it was influenced by attention. A greater attention distribution caused larger differences between normal and amblyopic eyes. Furthermore, temporal deficits also seemed to affect binocular conditions, since a slightly larger interocular imbalance was recently reported in mid-to-low temporal frequencies by Kosovicheva et al. [72], although stereopsis was unaffected. These findings are supported by other authors, such as Bonneh et al. [73], who reported a higher time latency when a stimulus is shown to an amblyopic eye compared to a non-amblyopic eye, as well as by the worse response to flickering stimulus compared to steady stimulus in amblyopes that was described by Ruddock et al. [74].

Thus, spatial and temporal frequency should be adapted to the individual features of amblyopic eyes, using low spatial frequencies and steady stimuli at the beginning of visual therapy in amblyopia, as the evidence suggests, because this is easier to perceive for amblyopic patients. As the patient improves, higher frequencies and flickering stimulus can be used. Likewise, flickering stimulus can be used to maintain patient attention [71].

#### 3.3.2. Contrast Sensitivity and Luminance

Monocular contrast sensitivity loss in amblyopia has been widely reported by many authors—even in treated amblyopes who achieved 20/20 visual acuity in clinical and experimental studies [50,75,76,77,78]. In addition, contrast reduction increases blur and crowding perception in monocular conditions [79]. Furthermore, a functional magnetic resonance imaging (fMRI) study showed a deficit in the contrast sensitivity in the V1 and extra-striate cortex, mainly in high contrast, due to a neural deficit where the loss of function is greater for parvocellular pathway activity [80]. Therefore, there is an evident and well-documented monocular deficit in contrast sensitivity in amblyopia. The affectation of this deficit to binocular vision in amblyopia should also be considered. The binocular summation of contrast sensitivity in amblyopia is an important factor to consider during amblyopia treatment. Pardhan et al. [81] reported that in all amblyopes, binocular summation decreases until inhibition is produced, as the average interocular differences in contrast sensitivity increase. Binocular inhibition was observed especially in middle to high frequencies, and seemed to be higher in strabismic amblyopia. This could be the reason for the report of better vision with the non-amblyopic eye by some amblyopes compared to the use of both eyes together. However, the presence of sensory fusion and stereopsis should also be considered so as to understand this effect in daily practice. According to this, optimal refractive correction and amblyopia treatment is supposed to reduce interocular differences in contrast sensitivity and, therefore, improve binocular summation.

Regarding the perception of deficits in luminance, it has been reportedly observed in dichoptic conditions, which suggests that interocular suppression has an impact on luminance processing [79]. Therefore, treatment with high contrast stimuli may be the best option during monocular training at the beginning of the vision therapy, since it is related with a more affected pathway and is easier to perceive for the eye. Furthermore, to fit the luminance during dichoptic therapy so as to decrease crowding and interocular suppression seems to also be an appropriate option. Orientation and contrast detection tasks can be used for training contrast sensitivity loss in amblyopia, since they were shown to improve letter recognition and contrast sensitivity [82].

#### 3.3.3. First- and Second-Order Stimulus

First-order stimuli are luminance-defined, mainly processed in the striate cortex (V1), and are related to orientation and spatial frequency perception. However, second-order stimuli are contrast-defined, but are also related with movement and orientation. Second-order stimuli are processed in V2, V3 and V5, that is, the extra-striate cortex [83]. Some authors have shown higher crowding in the second-order stimulus than the first-order when the target and flankers are of the same order [84]. Psychophysical experiments have also shown that training with first-order stimulus improves performance in detection tasks with luminance-defined letters, and the improvement is transferred to a second-order detection task with contrast-defined letters, but this does not occur in reverse [85].

#### 3.3.4. Color and Achromatic Stimuli

Amblyopes have shown deficits in processing color vision measured by neuroimaging, blood oxygen level-dependent (BOLD) activation and psychophysical tests. Bradley et al. [86] reported that in amblyopes, a loss of chromatic contrast sensitivity coincides with luminance contrast sensitivity. This means that contrast sensitivity decreased as spatial frequency increased in psychophysical experiments, while there was no difference in color discrimination in conventional clinical testing when low spatial frequencies were used [86]. These results have been supported by the most recent developments. Hess et al. [87] described a reduced cortical response to chromatic stimuli in the lateral geniculate nucleus (LGN), V1, V2, V3, VP, V3a and V4 in amblyopes measured with fMRI and BOLD signal. The dominant eye of amblyopes has shown a statistically better response for chromatic stimuli than achromatic, especially for the red/green (R/G) signal, while the amblyopic eye has shown an overall decreased chromatic response [87]. It is interesting to highlight the selective loss response in the L/M opponent cone signal measured in LGN, while in the striate and extra-striate cortex the lower response was reported in both the L/M opponent cone and the S cone response. These findings, based on experimental research, lead us to suggest the use of achromatic stimuli for the initial stages of vision therapy in order to induce larger neural responses, since color and luminance processing are physiologically separated. Color stimuli might be added in advanced stages of treatment for completing the therapy, although there is no empirical evidence about how chromatic stimuli can affect vision recovery, and this should be investigated in depth in the future. It is important to remark that these deficits in color processing cannot be confused with impairments in color perception, such as daltonism, since color perception is normal in amblyopic subjects.

#### 3.3.5. Figure–Ground Segregation

Visual deficits and crowding in amblyopes do not inhibit their ability to segregate an “E” from a mask made of spatial frequencies [88]. However, there is a deficit in figure–ground segregation when a light spot is used as a target and a random-dot noise as a mask [89]. Figure–ground segregation tasks also showed a decrease in temporal frequency resolution in amblyopic eyes when compared with the fellow eye, according to the results of Spang and Fahle [90]. An interesting aspect of the results of these authors is that spatial frequency does not seem to be the cause for this decrease, since similar temporal thresholds were reported when the spatial resolution was deteriorated in dominant eyes using plus lenses, although a small correlation between visual acuity and a longer temporal delay in amblyopic eyes was found. These results are like those reported by Wang et al. [91], who observed a worse performance in motion-defined and texture-defined tasks in amblyopes, although there were no significant differences in the global motion task. Therefore, amblyopia is a spatial and temporal disturbance that shows some deficits in figure–ground segregation, which should be considered during vision training due to its relationship with the presence or not of masking stimuli. Nonetheless, further research is needed in order to determine the underlying neural mechanisms and how they affect amblyopic subjects, in order to optimize the visual training concerning these issues.

Until now, some authors have proposed interesting neural network architectures to explain the theoretical basis of this process, but without systematic evaluation against human perception in terms of figure–ground rules and their combinations. fMRI studies have shown that illusory contours elicit responses in the Lateral Occipital Complex and that salient regions of the figure activate this area, but it still remains unknown which cues are used by the brain to detect them and how these image cues are computed. In 2008, Domijan and Šetić [92] proposed a neural model based on the interaction between the ventral and the dorsal processing stream, which can account for classical and recent principles of figure–ground organization. However, their results contradict the physiological findings about border ownership responses in V2, so further research in this field is still required [93].

#### 3.3.6. Signal–Noise

In the presence of noise, the stimuli detection efficiency decreases with the increase in spatial frequency, and therefore internal noise seems to be stimulus-dependent. Furthermore, amblyopes show an internal noise which is partially dependent on external noise. Therefore, there is a decreased efficiency in stimuli perception when external noise and/or spatial frequency increase [94,95,96,97]. Some authors suggest there are compensating mechanisms which are focused on low spatial frequencies in amblyopic eyes [98]. Motion and orientation integration are affected by the signal–noise ratio in amblyopia, although this fact is not justified by the low visibility of amblyopic eyes due to the deficits of V1. Therefore, it is suggested that the extra-striate cortex plays an important role in these alterations, since there are areas, such as V5, that are affected by amblyopia [99]. Based on these findings, visual training in amblyopia may start with minimum noise level stimuli in order to facilitate the amblyopic eye perception, and then higher levels of noise can be used as the amblyopia eye improves.

#### 3.3.7. Steady and Dynamic Stimulus

Motion perception is rarely assessed clinically, but there are motion perception and oculomotor deficits reported in amblyopia [32] that should be considered for a better understanding of this ocular disorder and its management. Anisometropic amblyopia has shown the same pattern of cortical activation with motion displacement stimuli in fMRI, but this pattern has shown a lesser extent of activation than controls, and this fact was even worse in strabismic amblyopia [100]. It should be considered that global motion sensitivity depends on the spatial and temporal parameters of the motion stimulus. Amblyopic children have shown deficits in finer spatial displacements, regardless of temporal parameters, which typically mature later during development [101,102]. Furthermore, speed motion perception is not affected by the reduction in perceived luminance of the amblyopic eye in dichoptic conditions [103], but there are some motion-defined form deficits in amblyopia that affect both amblyopic and fellow eyes, possibly due to the altered binocular mechanisms [104]. In terms of treatment, there is little information about how spectacles, occlusion or vision therapy affect motion perception. Giaschi et al. [105] did not find an improvement in motion perception after occlusion therapy, while Chen et al. [106] reported that baseline differences in motion responses between amblyopes and controls are solved after treatment. Birch et al. [104] reported some benefits in motion perception after binocular therapy. Based on these facts, steady or long and slow-motion stimuli may be a good option for starting visual training in amblyopia.

### 3.4. Summary Table of Parameters and Visual Tasks According to the Type of Stimulus

Table 2 summarizes suggestions derived from the scientific evidence concerning which parameters could be modified according to the type of stimulus for vision training in amblyopia.

Based on the literature above, some recommendations are provided for visual tasks and the initial parameters of the stimulus that should be used in vision training for amblyopia, which are summarized in Table 3.

### 3.5. Psychophysical Methods for Stimuli Presentation

Contrast sensitivity, stimulus size, and spatial and temporal frequencies are parameters that should be modified in an adequate range for every single amblyopic patient during visual training, the role of the psychophysical method used for that purpose being crucial. Clinical studies have shown the utilization of different methods for controlling stimulus presentation in visual trainings of amblyopic eyes, such as the staircase method [47] that uses the same sizes of intervals during training. Additionally, there are experiences reported with the use of the forced-choice response method [67] that is mainly useful in discrimination and orientation tasks, as well as with the use of predefined interval increases not following a specific psychophysical methodology [15]. In addition, there are studies not mentioning or describing in detail this issue [13]. However, among the variety of psychophysical methodologies that are available, adaptative methods, such as the Best-PEST or QUEST, seem to be an appropriate option for sequential visual trainings in amblyopia, but this should be investigated further. For example, with the Best-PEST methodology, changes in the parameters of the stimuli are adapted to the patient, based on information that is being gathered during the training and following some specific rules [107]. Thus, the examiner is sure that the patient is performing the detection tasks in an adequate threshold range [107]. Likewise, a combination of Best-PEST and forced-choice methods might also be useful for amblyopia training treatment. In this way, with the Best-PEST method, exercises will use the appropriate threshold range during stimulus presentation to promote the visual improvement, and with the forced-choice method clinicians could check out objectively the progress of the patient according to the results of the visual tasks. More research is still needed on this issue, which is not considered as a relevant point in most of the research on visual training in amblyopia.

### 3.6. Type of Environment: Perceptual Learning, Dichoptic Stimulations, and Virtual Reality

Technological progress aside, the advances in amblyopia research led to the development of new ways to use psychophysical stimuli for stimulating amblyopic eyes, such as computer-based vision therapy and the use of virtual reality devices. Computer-based perceptual learning consists of the use of software which applies the psychophysical stimuli to stimulate the amblyopic eye throughout the repetition of different visual tasks. On the one hand, perceptual learning with letters optotypes, Vernier’s stimuli and Gabor’s patches can be used in both monocular training, while the fellow eye is occluded with a patch [48], and binocular training, as part of binocular treatments such as dichoptic therapy and virtual reality devices. On the other hand, random-dot stimuli are specifically used for stereopsis training in the binocular environment. Computer-based dichoptic training uses psychophysical stimuli in a binocular environment, while the patient wears anaglyph red-green glasses and works with both eyes open [16]. In a dichoptic environment, the patient perceives a scene with some parts only viewed by the amblyopic eye and some other parts with the fellow eye, while a great part of the scene is being viewed with both eyes simultaneously. In this way, the treatment is focused on stimulating not only the amblyopic eye, but also the binocularity. For that purpose, the contrast and luminance of the fellow eye image can be reduced for avoiding interocular suppression, as reported in randomized clinical trials [108,109]. If there is suppression in the amblyopic eye, the patient would miss the part of the scene only perceived by this eye and would not be able to finish the exercise. Therefore, the use of dichoptic environments also provides feedback to maintain binocular fusion during training sessions.

Finally, virtual reality head-mounted displays (VR-HMD) can be a promising and innovative option for amblyopia treatment, as was recently reported [44]. These devices allow a clinician to apply psychophysical stimuli with a wide range of adjustable parameters in both monocular and binocular environments. Therefore, the amblyopic eye can be stimulated and, at the same, the fellow eye can be occluded or penalized to choose exercises with or without binocular conditions. Of course, virtual reality has pros and cons. The main advantage is related to the possibility of pitching the treatment as a funny video game for maintaining patient attention during training sessions, and the benefits of simulating three-dimensional environments for hand-eye coordination task [110].

Playing video games has been associated with increased cortical thickness and regional gray matter volume in different brain areas, such as the left dorso-lateral prefrontal cortex, left frontal eye field, hippocampal formation, insula or cerebellum [111], which could have great implications for amblyopic children in terms of presenting visual stimuli for rehabilitation purposes. This evidence indicates that integrating perceptual learning and dichoptic training tasks into a video game dynamic could provide more relevant and long-lasting clinical benefits to the patients than when these stimuli are presented on a computer screen without a gamification approach [112,113].

In contrast, it should be noted that the use of VR-HMD could have some impact on ergonomics [114], and accommodative and vergence response [115], which can cause some discomfort and mild symptoms to the patients. Therefore, the security and aftereffects of VR-HMD should be further investigated and reported as a part of the forthcoming studies on vision therapy.

## 4. Discussion

Amblyopia research has experienced considerable progress in the last few decades, providing clinicians with more knowledge about the neural basis of the amblyopic visual system and new therapeutic approaches. However, there is a wide heterogeneity in the methodology of vision therapy treatment for amblyopia, according to the scientific literature [8].

Understanding the neural mechanisms of amblyopia is crucial for designing adequate stimuli in vision therapy allowing an effective treatment based on perceptual learning, dichoptic training, or virtual reality. There are many studies about amblyopia vision therapy with well-described methods [9,15,49], but not all of them report a detailed description of how the stimuli used were selected and the exact neural mechanisms involved, apart from the reduced contrast sensitivity of the amblyopic eye or binocular vision imbalance [10,12,13]. Thus, visual deficits and neural mechanisms are described above to provide extensive information about this issue and to unify criteria for an appropriate evidence-based clinical practice in amblyopia vision therapy.

The main strength of this review is that it is the first to gather information specifically focused on stimuli features and psychophysics methods for amblyopia vision therapy. Therefore, this article can be used as a guideline for designing new treatments based on perceptual learning, dichoptic therapy, or VR devices. However, there are some limitations that should be considered. First, many of the included studies are psychophysical experiments and consequently the results are associated with a certain degree of subjectivity, since the response depends on the subject’s performance during experiments. This fact could be solved in future with further research using imaging or neurophysiological techniques for correlating neurological findings with psychophysical experiments and their impact on amblyopia recovery [116]. Second, the usual clinical practice does not, as yet, include specific tests to assess all the amblyopic visual deficits. Thus, there is not enough evidence about how all these deficits can change with vision therapy. Third, the recommendations provided for initial parameters and types of stimuli are based on the results of this narrative review, but still there is not enough peer-reviewed literature concerning the impact of some of them on vision restoration in amblyopia, such as the color of the stimulus, the use of first- or second-order stimulus, and motion. For this reason, further research is needed to obtain empirical evidence which corroborates or refutes these findings. To this effect, the implementation of computer-based and VR-HMD treatments might allow practitioners to obtain more detailed information about visual deficits from treated patients based on the result of the training tasks.

## 5. Conclusions

To sum up, vision therapy with perceptual learning, dichoptic training and VR-HMD seems to be a promising option for promoting visual recovery and rehabilitation in amblyopia, or accelerating the treatment period when combining it with patching. However, it is essential to adapt the stimuli to the patient’s individual baseline features according to the spatio-temporal frequency, contrast sensitivity, luminance, first- or second-order, color, figure–ground segregation, signal–noise ratio, and motion. Furthermore, stimuli can be used in both monocular and binocular training using a computer or VR-HMD. These parameters together with the use of an adaptative psychophysical method could be the optimal way to stimulate the amblyopic visual system, as the scientific literature suggested. However, this fact should be further supported with well-designed studies that include new psychophysical experiments as well as MRI studies and clinical trials with control groups.

## Figures and Tables

**Table 1:** Summary of the main outcomes obtained and the type of stimuli used in different clinical studies that have been revised in the current review.

Authors	Design	*N*	Type of Amblyopia	Enviroment	Stimulus	Population	Main Outcome
Jia et al., 2018 [50]	NRSI	19	Aniso	Perceptual learning	Gabor’s patch	Young adult	AUCSF improved from 8.41 ± 1.09 to 15.48 ± 1.61 VA increased about 2 lines
Liu et al., 2018 [51]	NRSI	13	9 aniso, 1 strab, 3 mixed	DT	Gabor’s patch	Young adults	Stereopsis improved 26.5% ± 6.9%
Avram et al., 2013 [48]	Case series	5	Aniso	PL	Letter optotypes	Children	Significant improvement of VA and CS after training
Chen et al., 2016 [52]	NRSI	13	Aniso	PL	Gabor’s patch	Teenagers and young adults	VA increased 1.64 ± 0.06 lines CSF improved significantly
Gambacorta et al., 2018 [9]	NRSI	29	Aniso	PL and DT	Gabor’s patch	Teenagers	VA improved 0.1 ± 0.03 LogMAR after 10 h of training
Zhang et al., 2013 [49]	Retrospective	341	Aniso, strab, ametropic and mixed	PL	Gabor’s patch Letter optotype	Children	Improvement in VA with PL was similar to with patching
Levi et al., 1997 [53]	NRSI	11	4 aniso, 4 strab, 3 mixed	PL	Vernier’s stimulus	Adults	Improvement in Vernier acuity
Portela-Camino et al., 2018 [16]	RCT	32	2 aniso, 18 strab, 10 mixed, 2 isoametropic	PL	RDS	Children	Stereopsis increased about 50% with RPST and 46.42% with Wirt circles
Martín-González et al., 2020 [54]	NRSI	16	2 aniso, 8 strab, 4 mix, 2 isoametropic	PL	RDS	Children	Stereopsis experienced a significant improvement

VA: visual acuity; NRSI, non-randomized studies of intervention; AUCSF: area under the contrast sensitivity function curve; DT: dichoptic training; PL: perceptual learning; CS: contrast sensitivity; CSF: contrast sensitivity function; RCT: randomized clinical trial; RDS: random-dot stereogram; RPST: Randot Preschool Stereoacuity Test: ***N***: number of eyes; LogMAR: logarithmic minimum angle of resolution.

**Table 2:** Parameters that could be modified according to the type of stimulus in vision training for amblyopia.

	Spatial and Temporal Frequency	Contrast Sensitivity and Luminance	First- and Second-Order	Color	Figure–Ground Segregation	Signal–Noise	Motion
Letter optotypes	Yes	Yes	Yes	No	Yes	Yes	Yes
Gabor´s patch	Yes	Yes	No	No	Yes	Yes	Yes
Vernier´s stimulus	Only temporal frequency	Yes	No	No	No	No	Yes
RDS	No	Only luminance	Yes	Yes	No	Yes	Yes

In “Color” column, “Yes” means color stimulus and “No” means achromatic. RDS: random-dot stereogram.

**Table 3:** Summary of the recommendations for the selection of visual tasks and the initial parameters of the stimulus that should be used in vision training for amblyopia according to the scientific evidence revised.

Stimulus	Initial Parameters	Visual Tasks
Letter optotypes	1. Low spatial frequency and prolonged exposure 2. Maximum contrast 3. First order achromatic and steady stimulus without noise or masking	Letter recognition with and without crowding Orientation discrimination
Gabor’s patch	1. Low spatial frequency and prolonged exposure 2. Maximum contrast 3. Achromatic and steady stimulus without noise nor masking	Orientation discrimination
Vernier’s stimulus	1. Prolonged exposure 2. Maximum contrast 3. Achromatic and steady stimulus without noise nor masking	Continuity discrimination
RDS	1. Decreased luminance in fellow eye for allowing binocular vision 2. First order achromatic and steady stimulus 3. Neither masking nor noise	Objects recognition in stereoscopic conditions

RDS: random-dot stereogram.
